# Assessing Social Participation Among Kidney Transplant Recipients Using PROMIS Computer Adaptive Testing

**DOI:** 10.1016/j.ekir.2025.05.023

**Published:** 2025-05-19

**Authors:** Maria G. Pucci, Mowa Ayibiowu, Jad Fadlallah, Aghna Wasim, Nathaniel Edwards, Madeline Li, Doris Howell, Susan Bartlett, John D. Peipert, Samantha Anthony, Istvan Mucsi, Janine Farragher

**Affiliations:** 1 Ajmera Transplant Centre, University Health Network, Toronto, Ontario, Canada; 2 Psychosocial Oncology, Princess Margaret Cancer Centre, Toronto, Ontario, Canada; 3 Princess Margaret Cancer Research Institute, Toronto, Ontario, Canada; 4 Centre for Outcomes Research and Evaluation, Research Institute of the McGill University Health Centre, Montreal, Quebec, Canada; 5 Division of Clinical Epidemiology, Department of Medicine, McGill University, Montreal, Quebec, Canada; 6 Department of Medical Social Sciences, Northwestern University Feinberg School of Medicine, Chicago, Illinois, USA; 7 Northwestern University Transplant Outcomes Research Collaborative, Comprehensive Transplant Center, Feinberg School of Medicine, Chicago, Illinois, USA; 8 Child Health Evaluative Sciences, Research Institute, The Hospital for Sick Children, Toronto, Ontario, Canada; 9 Department of Medicine, University of Toronto, Toronto, Ontario, Canada; 10 Department of Occupational Science and Occupational Therapy, University of Toronto, Ontario, Canada

**Keywords:** kidney transplantation, PROMIS, social participation

## Abstract

**Introduction:**

Social participation is a valued aspect of quality of life among kidney transplant recipients; however, few validated measures exist to assess it. This study aimed to explore the reliability and validity of the Patient-Reported Outcomes Measurement Information System (PROMIS) Ability to Participate in Social Roles and Activities (PROMIS-SP), administered as a computer adaptive test (CAT), among kidney transplant recipients.

**Methods:**

This was a cross-sectional study involving a convenience sample of adult recipients from Toronto, Canada. Participants completed the PROMIS-SP CAT and legacy measures of social participation on an electronic data capture platform. Reliability of the PROMIS-SP CAT was determined using standard error of measurement (SEM) and test-retest reliability using intraclass correlation coefficient. Convergent validity was assessed by calculating Spearman’s correlation between the PROMIS-SP CAT and legacy measures, and construct validity was assessed using known group comparisons.

**Results:**

We recruited 284 participants with a mean (SD) age of 53 (14) years and a median of 5.5 years since kidney transplantation; 61% were male, 53% were White, and 30% had diabetes. The mean (SD) PROMIS T-score was 51 (9). Reliability (*r* = 0.93) and test-retest reliability (intraclass correlation coefficient = 0.97) of the PROMIS-SP CAT were excellent. Strong correlations were observed between PROMIS-SP CAT and social difficulty inventory (SDI) (rho = −0.65), SDI “everyday living” (rho = −0.68), and EuroQol-5 Dimensions-5 Levels (EQ5D5L) “usual activities” (rho = −0.66). PROMIS-SP CAT scores were significantly different between known groups in the expected direction.

**Conclusion:**

Our results support the validity and reliability of PROMIS-SP CAT among kidney transplant recipients, and suggest that this tool can be used to identify recipients with restricted social participation.

## Graphical abstract

**Figure:**
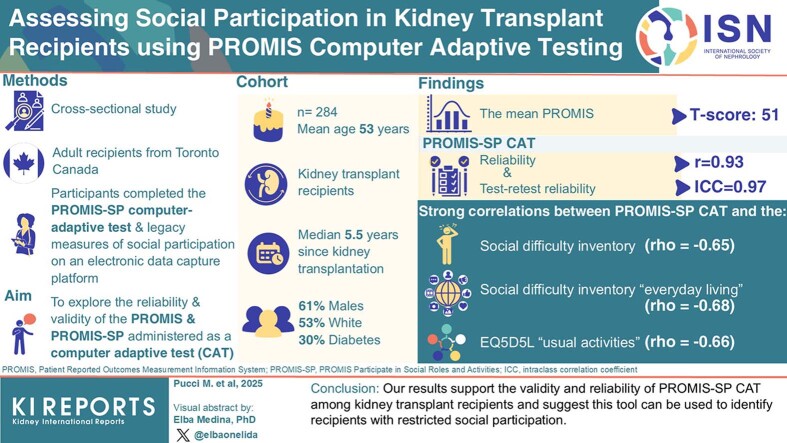

Kidney transplant recipients experience better survival and overall quality of life than patients on dialysis.^1^^,^^2^ Social participation is “a person’s involvement in activities that provide interaction with others in society or the community.”^3^ The global Standardized Outcomes in Nephrology– Kidney Transplant Initiative recently identified life participation, which includes social participation, as an important domain of health-related quality of life, and the need for robust measurement tools, as a top priority of patients and stakeholders.^4^

Compared with patients on dialysis, kidney transplant recipients typically experience fewer physical and mental symptoms such as fatigue, depression, and anxiety posttransplant, and are spared from the limitations imposed by dialysis (time burden and side effects).^5^, ^6^, ^7^, ^8^ Although this can increase their ability to participate in social activities,^9^^,^^10^ the side effects of immunosuppressive medications, infections, persistent chronic physical or psychological symptoms and comorbidities can limit social participation.^2^^,^^10^, ^11^, ^12^, ^13^, ^14^, ^15^ Patient-reported outcome measures, such as the SDI,^16^^,^^17^ the EQ5D5L,^18^^,^^19^ and the Kidney Disease Quality of Life Instrument-36 Item Short Form (KDQOL-36)^20^^,^^21^ have previously been used to evaluate social participation among kidney transplant recipients^2^; however, these tools have limitations. For example, some are broader quality-of-life measures that only include a few questions about social participation,^17^^,^^22^ limiting their ability to capture the full range of limitations kidney transplant recipients might experience.^2^ These measures can also be lengthy and tend to have relatively high floor and ceiling effects, indicating that they are not able to capture social participation at the high or low ends of the measurement spectrum. A feasible, valid, and reliable measure of social participation is therefore needed for the kidney transplant recipient population.

The PROMIS developed reliable and precise tools to assess physical, mental, and social domains of health,^23^^,^^24^ including the PROMIS-SP, which offers several potential advantages over other social participation measures. First, it has undergone a robust process of instrument codevelopment with patients and other stakeholders.^23^^,^^24^ Second, its validity and reliability have been demonstrated in the US general population, and in several chronic medical conditions.^25^, ^26^, ^27^, ^28^, ^29^ Third, PROMIS measures were developed using item response theory, and therefore can be administered both as fixed-length short forms, or CATs.^30^^,^^31^ CATs use score estimation algorithms to tailor administered items to the respondent, avoiding the need to complete irrelevant items, and minimizing the number of items the respondent must answer to generate a valid estimate.^32^^,^^33^ PROMIS CATs also have minimal floor and ceiling effects, ensuring reliability over a broad range of function level or symptom severity.^2^^,^^34^^,^^35^ As such, PROMIS-SP has recently been identified as a potential measure of social participation in the kidney transplant recipient population,^36^ and the psychometric properties of the PROMIS-SP short forms have been evaluated in several populations.^28^^,^^29^^,^^37^^,^^38^ However, the properties of the CAT version of PROMIS-SP have yet to be evaluated among kidney transplant recipients.

We therefore aimed to measure social participation among kidney transplant recipients using the PROMIS-SP CAT tool, and compared its psychometric performance with legacy participation measures used in this population.

## Methods

### Study Design and Population

We analyzed single-center, cross-sectional data on the PROMIS-SP CAT among kidney transplant recipients, collected as part of a larger study to assess the reliability and validity of PROMIS instruments among patients with different stages of chronic kidney disease. Adults (aged ≥ 18 years) kidney transplant recipients were recruited at the posttransplant clinics of the Ajmera Transplant Centre at the Toronto General Hospital. The studies were approved by the research ethics board at Toronto General Hospital (REB - #19-5097, REB - #15-9645), and all participants provided written informed consent before enrollment. Between November 2017 and May 2024, kidney transplant recipients were prescreened using chart review. Potentially eligible patients (kidney transplantation > 30 days before enrollment) were approached for screening and enrollment during scheduled outpatient posttransplant clinic visits, or through remote recruitment during the COVID-19 pandemic. Patients with severe acute conditions or visual impairments, who were not fluent in English or with incomplete or missing PROMIS-SP CAT were excluded (Supplementary Figure S1).

### Data Collection

#### Sociodemographic and Clinical Characteristics

Sociodemographic data (age, gender, marital status, education level, and racialized status) were self-reported. Socioeconomic status was assessed using the material resources dimension of the Ontario Marginalization Index, an area-level index that connects postal codes to census data. Each postal code in Ontario received a weighted score, which was ranked into quintiles, ranging from the least deprived (1) to the most deprived (5).^39^ For our analysis, these quintiles were grouped into low (quintiles 1 and 2), moderate (quintile 3), and high deprivation (quintiles 4 and 5). Clinical variables (comorbid conditions, blood hemoglobin, estimated glomerular filtration rate [eGFR], and serum albumin) were obtained from medical records. We used the 2021 eGFR equation that excludes race variables.^40^ Comorbid conditions were abstracted from the electronic patient record to calculate the Charlson Comorbidity Index.^41^

#### Questionnaire Administration

Participants completed all study questionnaires on iPads using an electronic data capture system (Data-Driven Outcomes System, Techna Institute, University Health Network, Toronto, Canada). If patients were off-site, a link to study questionnaires was sent via email using DADOS Connect, a secure University Health Network platform, which allowed participants to complete the questionnaires on their own devices. DADOS incorporated the HealthMeasures Assessment Center Application Programming Interface to administer the CATs. Participants completed 7 PROMIS domains (ability to participate in social roles and activities, physical function, fatigue, pain interference and intensity, depression, anxiety, and sleep disturbance), legacy questionnaires (SDI, EQ5D5L, and KDQOL-36), and a demographic questionnaire. All questionnaires were administered in English. Participants were also invited to retake the PROMIS-SP CAT questionnaire 3 to 14 days after the initial test to assess test-retest reliability.

#### PROMIS Ability to Participate in Social Roles and Activities (v2.0) Item Bank

The PROMIS-SP item bank consists of 35 items related to employment, social connectedness and relationships, and household activities.^42^ Items ask participants to reflect on perceived limitations (e.g., “I have trouble participating in recreational activities with others”). Each item is scored on a 5-point Likert scale (never, rarely, sometimes, usually, always).^42^ Responses are summed and converted into a standardized T-score, normalized to yield a mean (SD) score of 50 (10) for the US general population.^24^ A higher score reflects better social participation.^29^ For the PROMIS-SP CAT, a minimum of 4 items were administered, or until the SEM of 0.30 or lower was reached (corresponding to reliability of 0.9 or higher), or 12 items were completed, as per the parameters set by our team.^23^

#### Legacy Measures

The SDI-16 items (SDI-16) questionnaire was the primary legacy instrument used to assess convergent validity with the PROMIS-SP CAT, whereas the SDI “everyday living” subscale, EQ5D5L “usual activities” dimension, and KDQOL-36 question 12 were secondary legacy measures. The SDI-16 was developed and validated for use in routine oncology practice^17^ and has also been used in liver and kidney transplant recipient studies.^16^^,^^43^^,^^44^ It contains 16 items that inquire about difficulties in different domains of daily living activities (i.e., recreation, work, isolation, etc.).^17^ Each item is scored on a 3-point Likert scale ranging from 0 (no difficulty), to 3 (very much difficulty).^17^ SDI-16 scores range from 0 to 44, with higher scores indicating greater social difficulty.^17^^,^^45^ A cut-off score of ≥10 indicates potentially significant social distress.^17^^,^^45^ SDI-16 items are divided into 3 subscales: “everyday living,” “money matters,” and “self and others.”^17^ the “everyday living” subscale contains 6 items that inquire about an individual’s ability to carry out daily activities (i.e., recreation, independence, and personal care).^17^ Possible scores range from 0 to 16, with higher scores indicating greater difficulty.^17^

The EQ5D5L instrument assesses 5 dimensions, namely “mobility,” “self-care,” “usual activities,” “pain/discomfort,” and “anxiety/depression.”^22^ For each dimension, participants were asked to identify the statement that best applies (e.g., “I have no problems doing my usual activities”), and statements were rated on a 5-point Likert scale ranging from 1 (no problems), to 5 (extreme problem).^46^ The “usual activities” dimension asks participants to rate their ability to do usual activities such as work, leisure activities, family, study, and housework (e.g. “I have no problems doing my usual activities”).^22^

KDQOL-36 is a health-related quality-of-life measure that contains the generic SF-12 quality of life items, and 3 kidney-specific scales, including the “burden of kidney disease,” “effects of kidney disease,” and “symptoms/problems.”^47^ Item 12 of KDQOL-36 (KDQOL-36-Q12) specifically addresses social participation (i.e., During the past 4 weeks, how much of the time has your physical health or emotional problems interfered with your social activities [like visiting with friends, relatives, etc.]?). Responses are scored on a 5-point Likert response scale, ranging from 1 (all of the time) to 5 (none of the time). A higher score indicates better social participation.^47^

### Statistical Analysis

All statistical analyses were performed using Stata version 15.1. Proportions and means (SD) were calculated for parametric data, and median (interquartile range) for nonparametric data. Missingness was < 15% for variables in Table 1. Reliability at the participant level was assessed using SEM over the range of PROMIS-SP T-scores. Reliability coefficients were generated from the SEMs (reliability = 1 − SEM^2^). The reliability coefficient ranges from 0 (no reliability) to 1 (perfect reliability), with 0.9 being an acceptable reliability for individual scores.^48^ The group-level reliability was also calculated (average reliability = 1 − [mean SEM]^2^),^23^ with 0.9 being an acceptable reliability for groups.^49^ Test-retest reliability was determined by calculating the intraclass correlation coefficient from a 2-way mixed effects model. Only participants who completed the retest questionnaire in ≤14 days were analyzed. Reliability is considered excellent when values are > 0.90, or good when values range from 0.75 to 0.89.^50^

**Table 1:** Baseline sample characteristics for total sample, kidney transplant recipients

Variable	Total Cohort (*N* = 284)	PROMIS-SP CAT T-score		*P*-value
		< 50 (*n* = 128)	≥ 50 (*n* = 156)	
Age, yrs; mean (SD)	53 (14)	54 (14)	52 (14)	0.169
Male gender, *n* (%)	173 (61)	69 (54)	104 (67)	0.028
Self-reported racialized status, *n* (%)				
White	151 (53)	64 (50)	87 (56)	0.191
Asian	68 (24)	37 (29)	31 (20)	
African, Caribbean, or Black	23 (8)	14 (11)	9 (6)	
Other	19 (6)	8 (6)	11 (7)	
Marital status, *n* (%)				
Single	60 (21)	21 (16)	39 (25)	0.040
Married/common-law	162 (57)	75 (59)	87 (56)	
Divorced/widows	32 (11)	20 (16)	12 (8)	
OMI material resources, *n* (%)				
Low deprivation	98 (35)	40 (31)	58 (37)	0.260
Moderate deprivation	63 (22)	34 (27)	29 (18)	
High deprivation	100 (35)	45 (35)	55 (35)	
Education, *n* (%)				
No schooling to high school degree	47 (17)	18 (14)	29 (19)	0.347
Bachelor’s degree	163 (57)	76 (59)	87 (56)	
Post-grad/professional degree	47 (17)	25 (20)	22 (14)	
Median yrs since transplant (IQR)	5.5 (0.87–13)	2.7 (0.5–10)	7.0 (2.3–14.2)	<0.001
Diabetes diagnosis, *n* (%)	85 (30)	51 (40)	34 (22)	<0.001
Comorbidity, *n* (%)				
CCI < 4	183 (64)	76 (59)	107 (69)	0.011
CCI ≥ 4	67 (24)	40 (31)	27 (17)	
Anemia, *n* (%)				
Hgb < 110 g/l	50 (18)	33 (26)	17 (11)	0.002
Hgb ≥ 110 g/L	216 (76)	90 (70)	126 (81)	
eGFR, *n* (%)				
< 30 ml/min per 1.73 m^2^	27 (10)	20 (16)	7 (4)	0.002
≥ 30 ml/min per 1.73 m^2^	243 (86)	103 (80)	140 (90)	
Serum Albumin, *n* (%)				
< 40 g/l	47 (16)	28 (22)	19 (12)	0.044
≥ 40 g/l	221 (78)	96 (75)	125 (80)	
PROMIS-SP CAT T-score; mean (SD)	51 (9)	44 (5)	58 (6)	<0.001
PROMIS depression CAT T-score; mean (SD)	49 (9)	53 (8)	45 (8)	<0.001
PROMIS pain interference CAT T-score; mean (SD)	39 (9)	43 (8)	36 (7)	<0.001
PROMIS fatigue CAT T-score; mean (SD)	51 (9)	56 (8)	47 (8)	<0.001

CAT, computer adaptive test; CCI, Charlson Comorbidity Index; eGFR, estimated glomerular filtration rate; IQR, interquartile range; OMI, Ontario Marginalization Index; PROMIS, Patient-Reported Outcomes Measurement Information System; SP, social participation.

Values are presented as mean (SD) or median (IQR) for continuous variables. For categorical variables values are presented as count (percentage).

Floor and ceiling effects were determined by calculating the percentage of observations that scored at the minimum and maximum observed T-scores within our sample, respectively. A ceiling or floor effect > 15% suggests poor measurement coverage.^51^

Convergent validity was assessed using Spearman’s correlation (to account for the skewed distribution of some of the variables) between PROMIS-SP CAT versus SDI, SDI subscale “everyday living,” the “usual activities” dimension of EQ5D5L, and KDQOL-36-Q12. Construct validity was further assessed using known-group comparisons. We compared PROMIS-SP CAT T-scores between preidentified groups based on demographic, clinical, and psychosocial attributes, which were expected to demonstrate different levels of social participation (according to the literature and clinical experience). Groups hypothesized to have lower mean T-scores (i.e., more impairment in participation) included older age (> 65 years),^52^^,^^53^ females,^53^ lower socioeconomic status,^54^ less education,^54^ moderate-to-severe depressive symptoms (PROMIS depression T-score ≥ 60),^54^^,^^55^ pain interference (PROMIS pain interference T-score ≥ 50),^56^ moderate-to-severe fatigue (PROMIS fatigue T-score ≥ 60),^57^ high comorbidity (Charlson Comorbidity Index ≥ 4),^52^ anemia (< 110 g/l),^52^ low eGFR (< 30 ml/min per 1.73 m^2^),^52^ and low serum albumin (< 40 g/l).^9^^,^^52^ To assess construct validity, we used a threshold of 75% agreement between our hypotheses and observed results as an indicator of sufficient construct validity.^58^ For these comparisons, we conducted a multivariable linear regression that adjusted for age and gender. We reported the age- and gender-adjusted means for the groups. Cohen’s *d* for each comparison was calculated by dividing the absolute difference in adjusted means derived from the multivariable linear regression model by the model’s residual SD (root mean squared error). This approach provides an effect size measure that accounts for covariates included in the regression model. Small, moderate, and large effect sizes were categorized based on Cohen's *d* values of 0.20 to 0.49, 0.50 to 0.79, and > 0.80, respectively.^59^ A *post hoc* contrast analysis using orthogonal polynomials was conducted to assess a linear trend in social participation T-score across the 3 categories of age, Ontario Marginalization Index material resources, and education. To discriminate between participants with potentially significant social difficulties, receiver operating characteristic (ROC) analysis was used with SDI ≥ 10 being the criterion against PROMIS-SP CAT. The area under the ROC curve was used to measure the ability of PROMIS-SP CAT to discriminate between people with versus those without potentially significant social difficulties.^17^^,^^45^ The area under the ROC curve values ≥ 0.8 generally indicate excellent discrimination.^60^

To identify a cut-off score that detects participants with and without social difficulties, a threshold analysis was conducted that included analyzing various data points on the ROC curve and calculating the Youden’s J Index.^61^ To identify response patterns that characterized respondents from different T-score ranges, a contingency table analysis was performed that described the last items administered to participants in relation to their mean T-score and the most common response when administered (Supplementary Table S2). Patterns of responses to last-items that were administered to people from different T-score ranges were also examined to further identify characteristic responses (Supplementary Table S3).

## Results

A total of 1284 participants were assessed for eligibility for the study; 597 participants declined, 72 participants were missed by the recruitment team, and 331 participants were excluded based on exclusion criteria (Supplementary Figure S1). A total of 284 participants were included in the analysis (Supplementary Figure S1). The mean (SD) age was 53 (14) years, 61% were male, and 53% were White. The mean (SD) PROMIS-SP CAT T-score was 51 (9). Baseline characteristics are reported in Table 1. PROMIS-SP CAT T-scores demonstrated near normal distribution and exhibited minimal floor effects (< 1%) compared with the legacy tools SDI (> 15%), EQ5D5L (> 15%), and KDQOL-36-Q12 (4%), with more scores at the ceiling (10%) than with the SDI and EQ5D5L (< 1%) (Supplementary Figure S2). The number of items completed on PROMIS-SP CAT ranged between 4 and 12, with a median (interquartile range) of 4 (0). Of the participant 237 (83%) completed ≤ 6 on PROMIS-SP CAT (Supplementary Figure S3).

**Figure 1:**
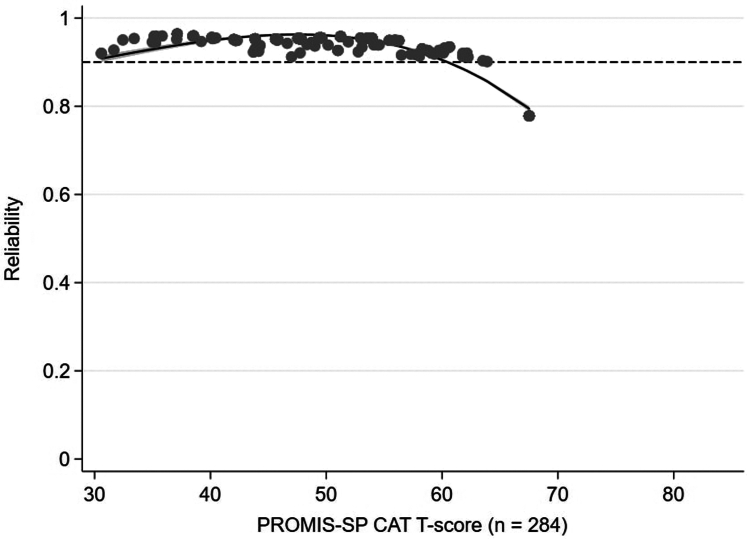
Reliability graph plotting reliability (1 − [standard error of measurement^2^]) against PROMIS Social Participation CAT T-scores for the entire cohort. Excellent reliability (0.9) is demonstrated by the dotted line. CAT, computer adaptive test; PROMIS, Patient-Reported Outcomes Measurement Information System; SP, social participation.

Individual score reliability was ≥ 0.90 for 85% of the overall sample across the PROMIS CAT SP T-score range of 31 to 64 (Figure 1). The mean SEM for the overall sample was 0.26, corresponding to an average reliability of 0.93, which is considered excellent. Seventy-six participants repeated the PROMIS-SP CAT after a median (interquartile range) of 7 (6) days. Test-retest reliability was excellent, with an average intraclass correlation coefficient of 0.97 (95% confidence interval: 0.96–0.98, *P* < 0.001).

**Figure 2:**
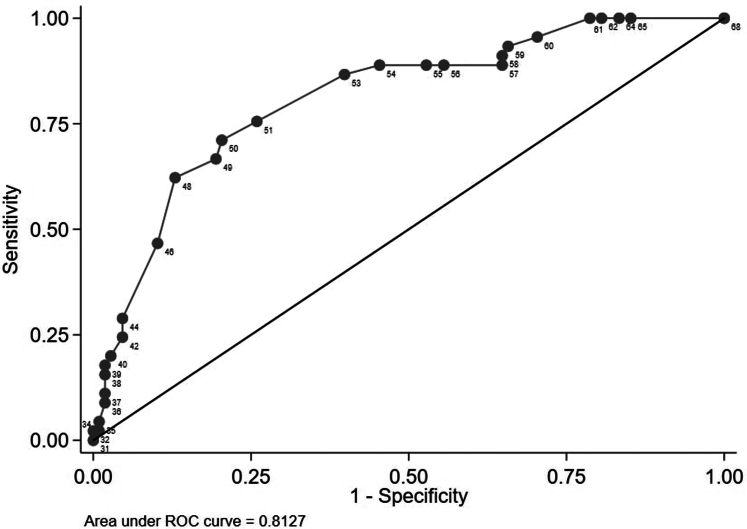
Box plot between PROMIS-SP CAT T-scores and EQ5D5L usual activities dimension (I have__ problems doing my usual activities) score for the whole sample. The boxes show the interquartile range (IQR) around the median (horizontal line), whereas the whiskers extend to 1.5 times the IQR. CAT, computer adaptive test; EQ5D5L, EuroQOL 5-Domain 5-Level; PROMIS, Patient-Reported Outcomes Measurement Information System; SP, social participation.

A strong negative correlation was observed between PROMIS-SP CAT and SDI-16 (rho = −0.65, *P* < 0.001) and with the “everyday living” subscale (rho = −0.68, *P* < 0.001) (Supplementary Figure S4). A strong correlation was also observed between PROMIS-SP CAT and EQ5D5L “usual activities” dimension (rho = −0.66, *P* < 0.001) (Figure 2). There was a moderate negative correlation observed between PROMIS-SP CAT and KDQOL-36-Q12 (rho = −0.58, *P* < 0.001) (Supplementary Figure S5).

In known-group comparisons, PROMIS-SP CAT T-scores were significantly different between groups based on sociodemographic variables such as gender and Ontario Marginalization Index material resources, but not age and education (Table 2). Mean (95% confidence interval) PROMIS-SP CAT T-scores were also significantly lower among participants with versus those without moderate-to-severe depression (44 [41–46) vs. 53 [52–54], *P* < 0.001), pain interference (43 [40–47] vs. 53 [52–54], *P* < 0.001), and moderate-to-severe fatigue (43 [40–45] vs 53 [52–54], *P* < 0.001). As for clinical variables, PROMIS-SP CAT T-scores were significantly lower among participants with more comorbidities, anemia, low serum albumin, and low eGFR (Table 2). Sufficient construct validity was demonstrated with 82% of the known-group comparisons in accordance with predefined hypotheses. There were large effect sizes for eGFR, depression, pain interference, and fatigue groups (Table 2). The *post hoc* contrast analysis identified no significant linear trend for age, Ontario Marginalization Index material resources, and education (Table 2).

**Table 2:** Known group comparison of age- and gender-adjusted means of PROMIS social participation CAT T-scores, obtained from linear regression models using the adjmean STATA command

Variable	Adjusted mean PROMIS social participation T-score (95% CI)	Cohen’s *d*	*P*-value	*P* for trend
Age (yrs)				
≤ 45 (*n* = 85)	52 (50–54)	Ref.	0.216	0.217
46–64 (*n* = 132)	52 (50–53)	0.01		
≥ 65 (*n* = 67)	50 (48–52)	0.20		
Gender				
Male (*n* = 173)	52 (51–54)	0.27	0.028	-
Female (*n* = 111)	50 (48–52)			
OMI material resources				
Low deprivation (*n* = 98)	52 (51–54)	Ref.	0.020	0.404
Moderate deprivation (*n* = 63)	49 (47–51)	0.38		
High deprivation (*n* = 100)	51 (49–53)	0.12		
Education				
No schooling to high school degree (*n* = 47)	53 (50–55)	Ref.	0.154	0.184
Bachelor’s degree (*n* = 163)	51 (49–52)	0.24		
Postgrad/professional degree (*n* = 47)	50 (48–53)	0.28		
Anemia				
Hb < 110 g/l (*n* = 50)	47 (45–50)	0.55	0.001	-
Hb ≥ 110 g/l (*n* = 216)	52 (51–53)			
eGFR				
< 30 g/l (*n* = 27)	46 (42–48)	0.80	<0.001	-
≥ 30 g/l (*n* = 243)	52 (51–53)			
Serum albumin				
< 40 g/l (*n* = 47)	48 (46–51)	0.41	0.013	-
≥ 40 g/l (*n* = 221)	52 (51–53)			
Charlson comorbidity index				
< 4 (*n* = 183)	52 (51–53)	0.42	0.004	-
≥ 4 (*n* = 67)	49 (47–51)			
Depression				
Mild/none (T-score < 60) (*n* = 205)	53 (52–54)	1.10	<0.001	-
Moderate/Severe (T-score ≥ 60) (*n* = 32)	44 (41–46)			
Pain interference				
T-score < 50 (*n* = 144)	53 (52–54)	1.10	<0.001	-
T-score ≥ 50 (*n* = 21)	43 (40–47)			
Fatigue				
Mild/none (T-score < 60) (*n* = 236)	53 (52–54)	1.40	<0.001	-
Moderate/severe (T-score ≥ 60) (*n* = 48)	43 (40–45)			

CAT, computer adaptive test; CI, confidence interval; eGFR, estimated glomerular filtration rate; Hb, hemoglobin; OMI, Ontario Marginalization Index; PROMIS, Patient-Reported Outcomes Measurement Information System.

The *P*-values corresponding to the result of the linear regression are presented by the values in the “*P*-value” column, whereas the significance of a linear trend is denoted by the values in the “*P* for trend” column. Cohen’s *d* values indicate the standardized effect size from the linear regression. For variables with > 2 groups, the reference group is labeled as “Ref.”

**Figure 3:**
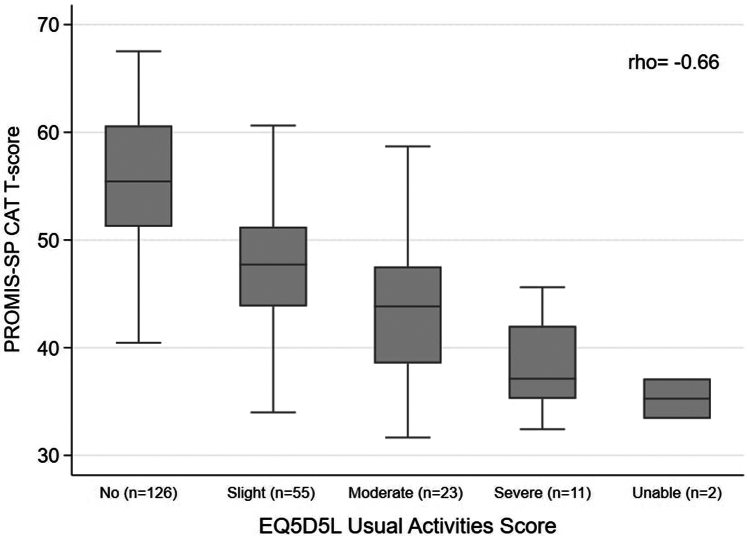
Receiver operating characteristic curve of PROMIS Social Participation CAT T-scores against social difficulty inventory (a SD-16 score of ≤ 10 represents clinically relevant social difficulties) for the entire cohort. The corresponding T-score values are displayed at each dot. CAT, computer adaptive test; PROMIS, Patient-Reported Outcomes Measurement Information System; ROC, receiver operating characteristic.

PROMIS-SP CAT discriminated well between participants with versus those without potentially significant social distress using an SDI ≥ 10 as a reference (area under the ROC curve: 0.81 [95% confidence interval: 0.7–0.9]) (Figure 3). To identify kidney transplant recipients with potentially significant social distress, a T-score of 49 (sensitivity = 71%, specificity = 80%) was the best cut-off in this dataset, based on the Youden’s J index (J = 0.51) (Supplementary Table S1). Based on the last items answered, those with no social difficulties (T-score > 49) were characterized by rarely having to limit regular activities with family or friends (Table 3). Furthermore, 35% of participants with a T-score < 50 reported experiencing at least some difficulty, because they responded with “sometimes” to at least 1 PROMIS-SP CAT question. Similarly, 32% of participants with T-scores between 40 and 45 and 29% of those in the 45 to 49 range also responded with “sometimes” to at least 1 PROMIS-SP CAT question.

**Table 3:** Synthesis of social participation patterns according to T-Score based on responses to last-Items administered – summary. For more details, see Supplementary Tables S2 and S3

PROMIS social participation T-score	Narrative summary of response patterns
≥ 60	*No trouble* socializing with family or friends, participating in recreational activities, or performing work tasks
50–59	Typically has no trouble with recreational activities, but *occasionally* might have to limit activities with family or friends
40–49	*Sometimes* has trouble socializing and doing activities with family or friends
30–39	*Usually* has trouble doing all of the work that is important to them, and doing activities with family and friends

PROMIS, Patient-Reported Outcomes Measurement Information System.

## Discussion

Although social participation is a highly important outcome to kidney transplant recipients, there are only a limited number of validated tools to measure social participation in this population. Whereas the Standardized Outcomes in Nephrology life participation initiative has recently developed a measure to capture the broader concept of life participation among kidney transplant recipients,^36^ PROMIS-SP can offer more nuance and insights into social difficulties experienced by kidney transplant recipients. We thus sought to compare the reliability and validity of the PROMIS-SP CAT assessment among kidney transplant recipients with legacy measures. PROMIS-SP CAT demonstrated excellent reliability among kidney transplant recipients across a wide range of T-scores and displayed excellent construct validity. The measure also demonstrated minimal floor effects compared with legacy measures, with only slightly higher ceiling effects than those measures. Most participants completed ≤ 6 items to obtain a reliable score. These results support the use of PROMIS-SP CAT as both a feasible and robust measure of social participation for kidney transplant recipients at the individual and group levels for research and clinical care.

In our study, PROMIS-SP CAT demonstrated excellent construct validity, which is consistent with previous research on the PROMIS-SP CAT in various other clinical populations.^28^^,^^29^ For example, our observation of lower mean PROMIS-SP CAT T-scores for patients reporting pain interference, fatigue, and depressive symptoms are consistent with several previous studies.^54^, ^55^, ^56^, ^57^ The differences observed in this analysis between groups defined by clinical variables (e.g., Charlson Comorbidity Index, serum albumin, eGFR, etc.) also aligned with expectations based on the previous literature,^9^^,^^52^ and surpassed the minimal clinically important difference of the PROMIS-SP reported in other populations.^62^ The strong validity demonstrated in this study aligns with previous work done by the Standardized Outcomes in Nephrology – Kidney Transplant project, and supports the notion that PROMIS-SP CAT can accurately assess social participation among kidney transplant recipients.

PROMIS-SP CAT also demonstrated excellent reliability across a wide range of T-scores, which suggests it is an appropriate measure for kidney transplant recipients with broadly different levels of social participation. Importantly, we found that the PROMIS-SP CAT exhibited strong test-retest reliability, similarly to previous results with PROMIS short-forms.^38^ In addition, our test-retest reliability findings are consistent with previous findings in the Dutch general population,^29^ and in people with rheumatoid arthritis.^28^ Similarly, PROMIS-SP CAT has demonstrated acceptable test-retest reliability among patients with chronic kidney disease not receiving renal replacement therapy.^63^

With most of our participants completing just ≤6 relevant items to obtain a reliable score, PROMIS-SP CAT offers advantages over other tools with respect to minimizing questionnaire burden. Although reliability was slightly reduced at very high T-scores (indicating very high social functioning), scores still demonstrated acceptable reliability and questionnaire burden in this range of scores, with a maximum of 12 items being administered. Our ongoing analysis demonstrates that the overall reliability of the assessment is not compromised even if the maximum number of items is limited at 6, suggesting the stopping rule of the CAT could be modified to further minimize questionnaire burden. PROMIS CAT offers various stopping rules to set when the questionnaire ends, offering versatility and very low question burden.^64^ Although this change would result in slight reduction of the reliability of scores at the extremes of the T-score range (T-score < 30 or > 65–70), it would not have significant impact on the clinical interpretation of the score. Overall, the strong reliability of PROMIS-SP CAT indicates it can be relied upon to provide stable and efficient measurement of social participation in the kidney transplant recipient population, with few administered items.

Our finding that the PROMIS-SP CAT exhibited minimal floor effects suggests it is a strong tool for capturing social participation among kidney transplant recipients with limited participation levels. This is critical for identifying clinically significant changes and estimating the efficacy of various interventions on social participation. Our results did suggest that PROMIS-SP CAT has higher ceiling effects than SDI and EQ5D5L, and it has previously been suggested that the ceiling effect of PROMIS-SP CAT item bank could be reduced in the future by adding items to reflect the experiences of those with higher levels of social participation.^65^ Nonetheless, the combined ceiling and floor effect of PROMIS-SP CAT was lower (< 15%) than that of the legacy instruments (> 15%). This supports its potential to accurately assess changes in social participation over time throughout the kidney transplant recipient journey, and through transitions between various stages and treatment modalities of chronic kidney disease.

Establishing a cut-off score for PROMIS-SP CAT is important for identifying individuals with impaired social participation who may benefit from further clinical assessment. For PROMIS T-scores, a distribution-based method is often suggested, typically considering 0.5 SD or 1 SD from the US general population mean T-score of 50 to indicate potentially significant symptom severity or functional impairment.^66^ Accordingly, thresholds of 45 and 40 could be used to identify mild and moderate-to-severe social participation difficulties, respectively. Our ROC analysis suggests a cut-off score of <49 for potentially significant social difficulties using the SDI ≥ 10 as a referent. A significant proportion of participants with T-scores in the 40 to 45 range, and in the 45 to 49 range, respectively, reported at least some difficulty with social participation, as indicated by their responses to PROMIS-SP items suggesting challenges at least “sometimes”.

In addition, our analysis of the last item participants answered confirmed that those with a T-score ≥ 50 rarely had social difficulties and those with a T-score < 50 indicated some limitations. Comparing the EQ5D5L “usual activities” dimension score with the PROMIS-SP CAT T-score, there were “no” problems in usual activities with a T-score ≥ 50. Moreover, participants with a T-score of approximately 45 indicate “slight” problems, which more closely aligns with the published PROMIS-SP cut-off.^67^ Therefore, depending on the desired level of sensitivity and the degree of suspicion for social difficulties, a cut-off score of 45 or 49 can be considered to identify patients with potentially significant social limitation for further assessment. The choice of an appropriate cut-off should depend on the context in which the PROMIS-SP CAT tool is used—whether to estimate the proportion of individuals facing any social difficulties, assess the severity of social participation limitations, or identify those in need of further evaluation and support. Further research is needed to determine patient-centered thresholds and clinical relevance through methodologies such as bookmarking or other qualitative, patient-centered approaches.

### Limitations

The study’s limitations include the use of convenience sampling, which might introduce selection bias. Our participants were from a single center and thus might not represent kidney transplant recipients from other centers, although our sample was large and ethnically and socio-economically diverse. Our study also excluded non-English speaking individuals because the study questionnaires were administered in English, which limits the generalizability of our findings to non-English speaking kidney transplant recipients. In addition, of the 284 participants, only 76 completed the repeat PROMIS-SP measurement, which might limit the generalizability of our findings specifically pertaining to test-retest reliability. Finally, because this was a cross-sectional study, we could not assess the responsiveness of PROMIS-SP over time. Our ongoing data collection will make possible future studies to establish the appropriateness of the PROMIS-SP CAT tool as an outcome measure.

## Conclusion

We demonstrated that PROMIS-SP CAT has strong reliability and validity with minimal floor and low ceiling effects and minimal questionnaire burden, distinguishing it from existing legacy tools. PROMIS-SP CAT may aid health care providers in identifying impairments in their patient’s social participation, a highly patient-valued domain of health-related quality of life.

## Disclosure

SB is the president of the PROMIS Health Organization and the coleader of PROMIS Canada. IM holds an unrestricted education grant from Astellas Pharma Canada, Paladin Labs Canada. All the other authors declared no competing interests.

## Supplementary Material

Supplementary File (PDF) **Figure S1.** Participant flow diagram for kidney transplant patients. **Figure S2.** Distribution of social participation and related measures (PROMIS-SP CAT T-scores, SD-16 scores, SD-16 “everyday living” subscale scores, EQ5D5L “usual activities” scores, and KDQOL-36 question 12). **Figure S3.** Number of PROMIS-SP CAT items answered relative to T-score. **Figure S4.** Correlation between PROMIS-SP CAT T-scores and SD-16 measures (total score and “everyday living” subscale). **Figure S5.** Relationship between PROMIS-SP CAT T-scores and KDQOL-36 question 12 scores. **Table S1.** PROMIS-SP CAT T-score cut-offs for identifying social distress using SD-16 as a reference. **Table S2.** PROMIS-SP CAT item administration frequency, response patterns, and mean T-scores. **Table S3.** PROMIS-SP CAT frequency of responses to last-items administered based on T-score range. STROBE checklist of items that should be included in reports of cross-sectional studies.

## Footnotes

Supplementary File (PDF)
**Figure S1.** Participant flow diagram for kidney transplant patients. **Figure S2.** Distribution of social participation and related measures (PROMIS-SP CAT T-scores, SD-16 scores, SD-16 “everyday living” subscale scores, EQ5D5L “usual activities” scores, and KDQOL-36 question 12). **Figure S3.** Number of PROMIS-SP CAT items answered relative to T-score. **Figure S4.** Correlation between PROMIS-SP CAT T-scores and SD-16 measures (total score and “everyday living” subscale). **Figure S5.** Relationship between PROMIS-SP CAT T-scores and KDQOL-36 question 12 scores. **Table S1.** PROMIS-SP CAT T-score cut-offs for identifying social distress using SD-16 as a reference. **Table S2.** PROMIS-SP CAT item administration frequency, response patterns, and mean T-scores. **Table S3.** PROMIS-SP CAT frequency of responses to last-items administered based on T-score range. STROBE checklist of items that should be included in reports of cross-sectional studies.
