# Supplementary material for: Assessing Social Participation Among Kidney Transplant Recipients Using PROMIS Computer Adaptive Testing
Source: Kidney Int Rep. 2025 May 19;10(8):2708–19. doi: 10.1016/j.ekir.2025.05.023 (PMC12348183; doi:10.1016/j.ekir.2025.05.023)
Supplement: Supplementary File (PDF) — Figure S1. Participant flow diagram for kidney transplant patients. Figure S2. Distribution of social participation and related measures (PROMIS-SP CAT T-scores, SD-16 scores, SD-16 “everyday living” subscale scores, EQ5D5L “usual activities” scores, and KDQOL-36 question 12). Figure S3. Number of PROMIS-SP CAT items answered relative to T-score. Figure S4. Correlation between PROMIS-SP CAT T-scores and SD-16 measures (total score and “everyday living” subscale). Figure S5. Relationship between PROMIS-SP CAT T-scores and KDQOL-36 question 12 scores. Table S1. PROMIS-SP CAT T-score cut-offs for identifying social distress using SD-16 as a reference. Table S2. PROMIS-SP CAT item administration frequency, response patterns, and mean T-scores. Table S3. PROMIS-SP CAT frequency of responses to last-items administered based on T-score range. STROBE checklist of items that should be included in reports of cross-sectional studies. [file mmc1.pdf]

Supplementary Tables and Figures

Figure S1: Participant flow diagram for patients with kidney transplantation.

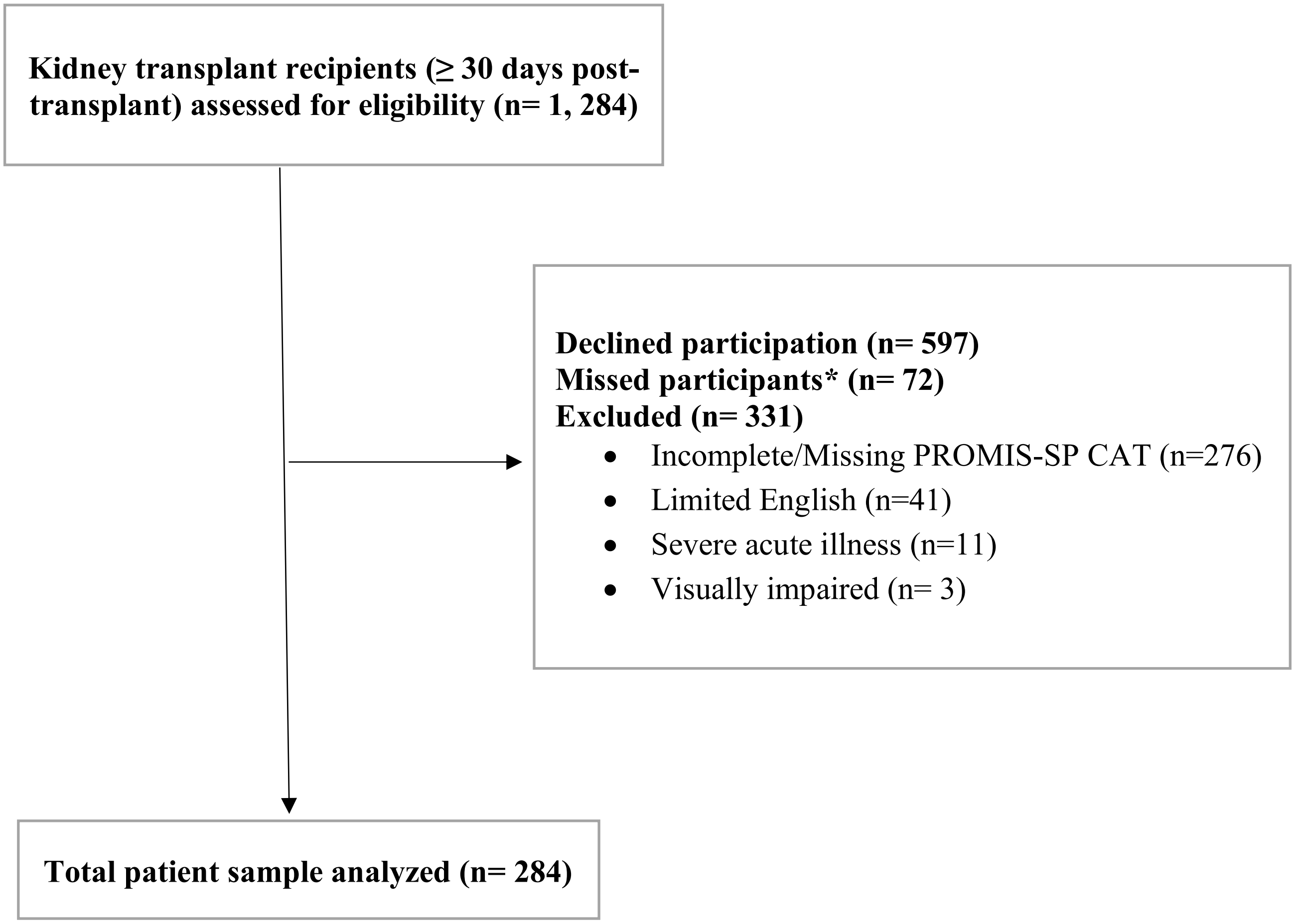

Footnote: Missed participants* are those who were eligible based on pre-screening assessments but were never approached in the clinic. This could be because they left the clinic before being approached or because there was no recruitment team available during their clinic visit.

Figure S2: Histogram plot presenting the distribution of (a) PROMIS Social Participation CAT T-scores, (b) SD-16 scores, (c) SD-16 “Everyday living” subscale scores, (d) EQ5D5L “usual activities” dimension scores, and (e) KDQOL-36 question 12 (During the past 4 weeks, how much of the time has your physical health or emotional problems interfered with your social activities [like visiting with friends, relatives, etc.]?)

2.a

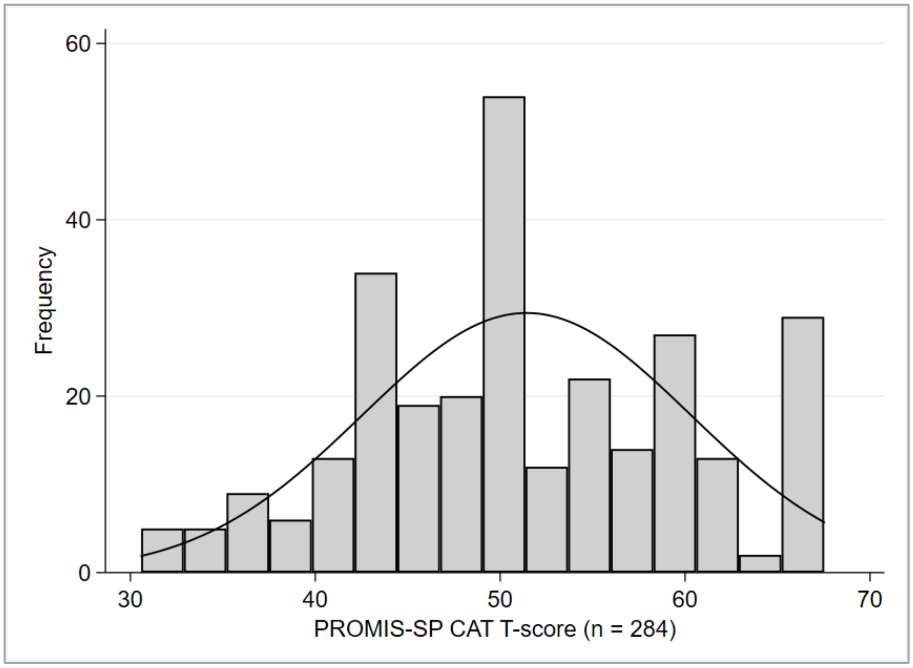

2.b

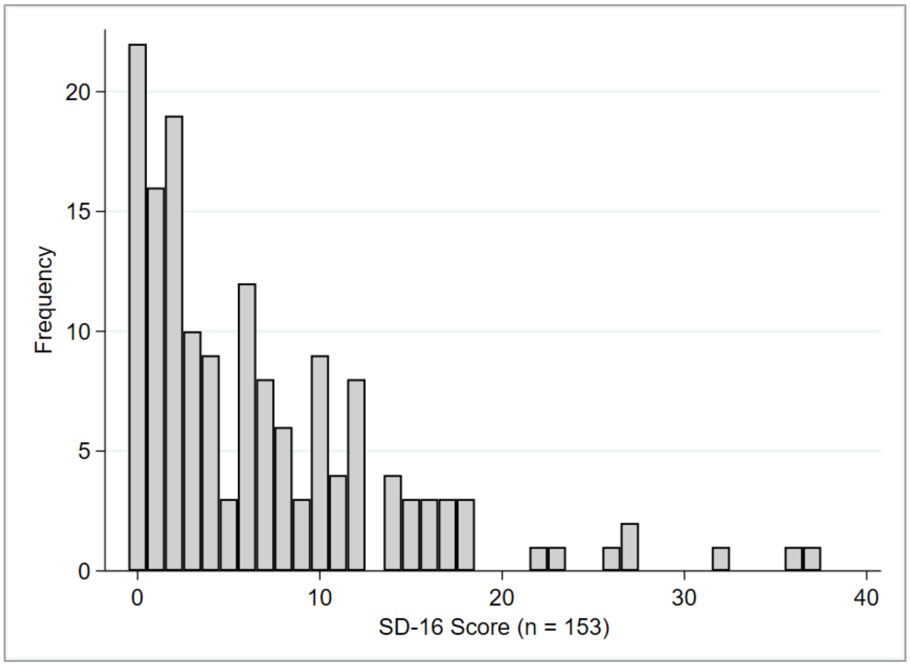

2.c

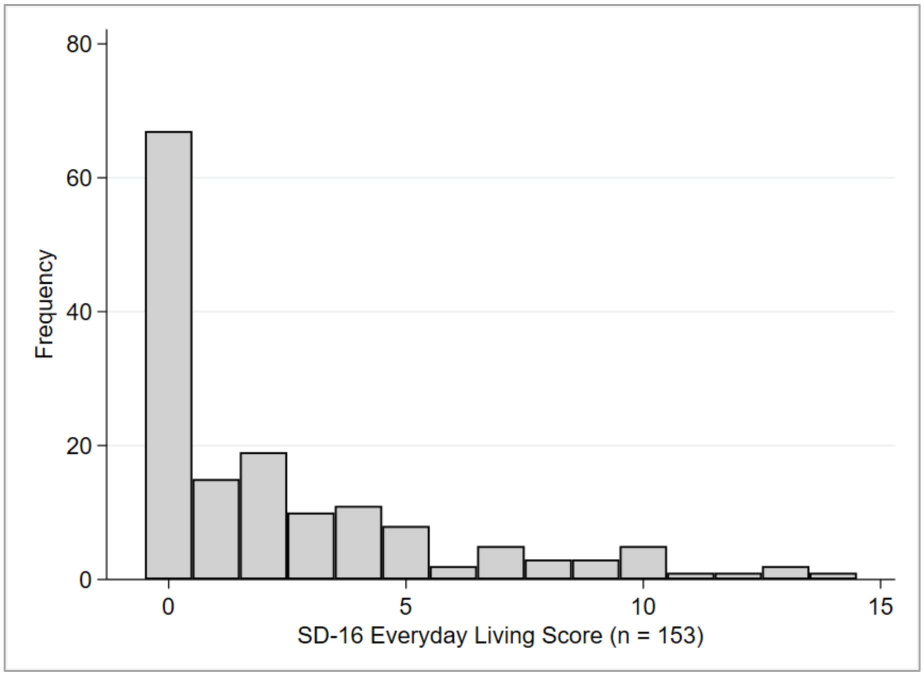
80
60
40
20
0
Frequency
0
5
10
15
SD-16 Everyday Living Score (n = 153)

2.d

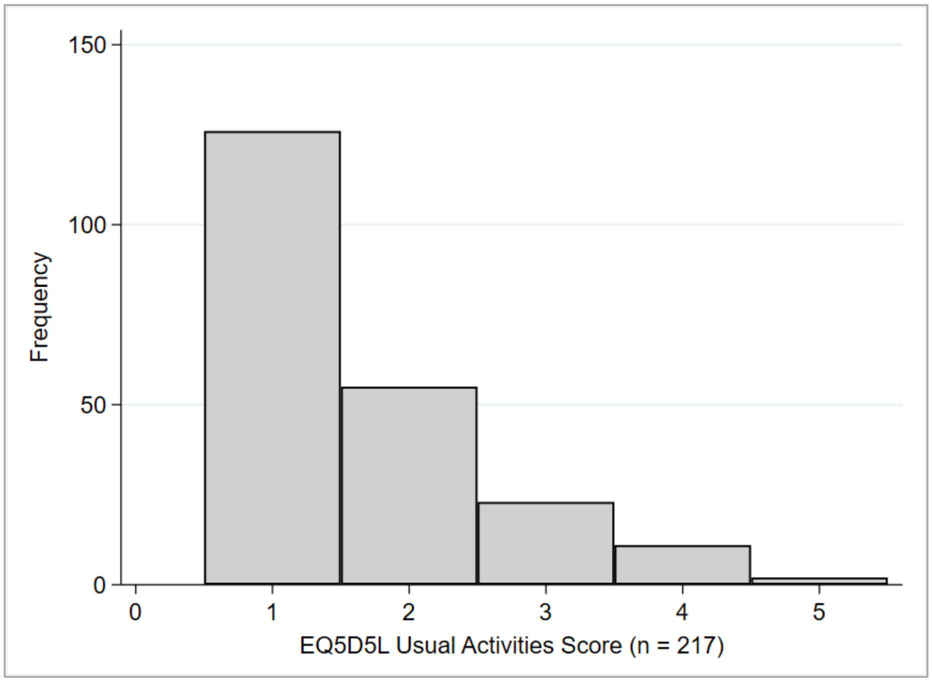
150
100
50
0
Frequency
0
1
2
3
4
5
EQ5D5L Usual Activities Score (n = 217)

2.e

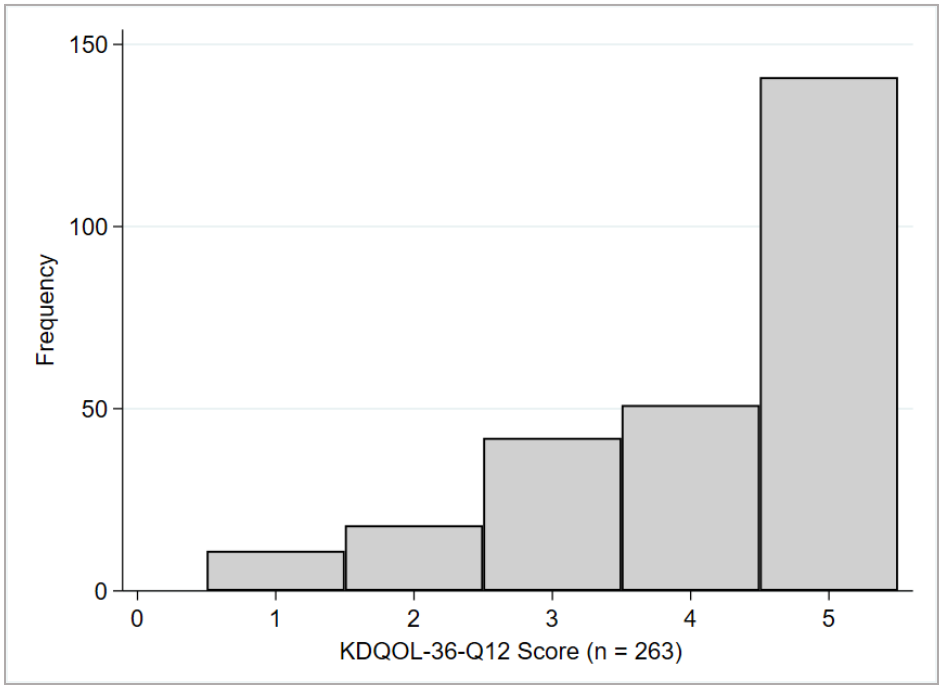

Footnote: Abbreviations: CAT, computer adaptive test; PROMIS, Patient-Reported Outcomes Measurement Information System; SP, social participation; SD-16, 16-item social distress; EQ5D5L, EuroQOL 5-Domain 5-Level; KDQOL-36, Kidney Disease Quality of Life Instrument-36 Item Short Form; Q12, question 12.

Figure S3: Total number of PROMIS-SP CAT items answered relative to T-score

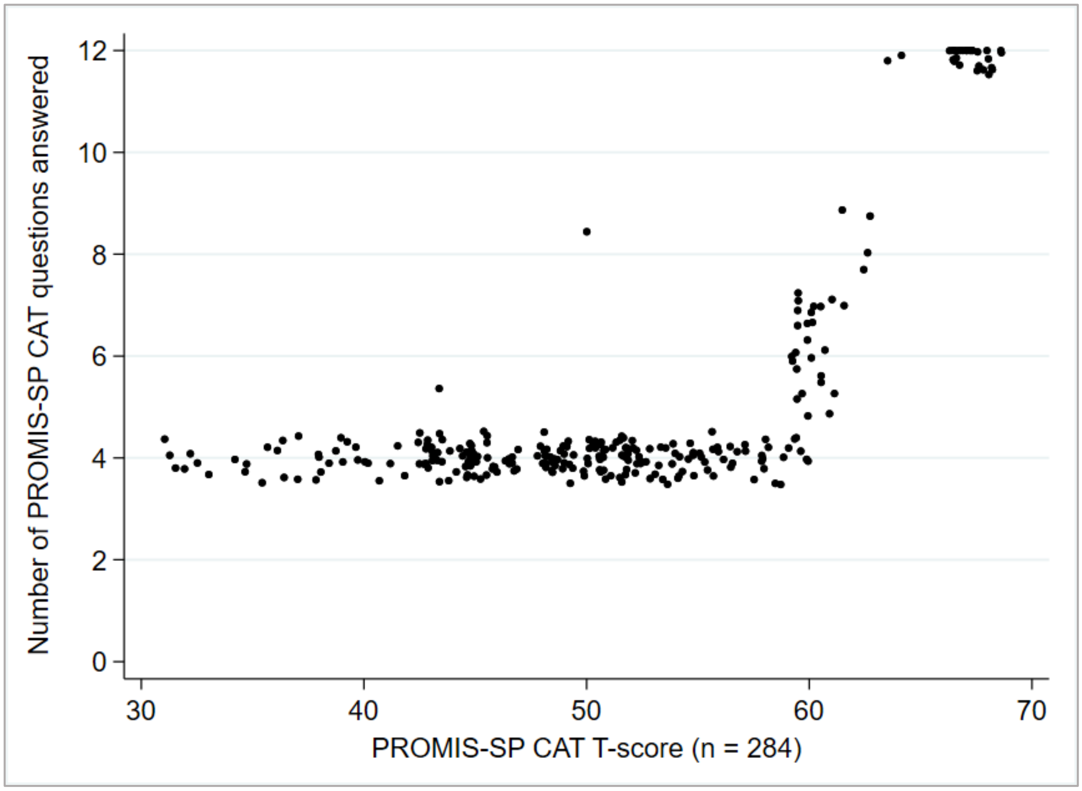

Footnote: Abbreviations: CAT, computer adaptive test; PROMIS, Patient-Reported Outcomes Measurement Information System; SP, social participation.

Figure S4: Scatter plot and linear fit plots (gray region corresponding to 95% confidence interval) between PROMIS-SP CAT T-scores vs. (a) SD-16 score and (b) SD-16 “everyday living” subscale score for the whole sample.

4.a

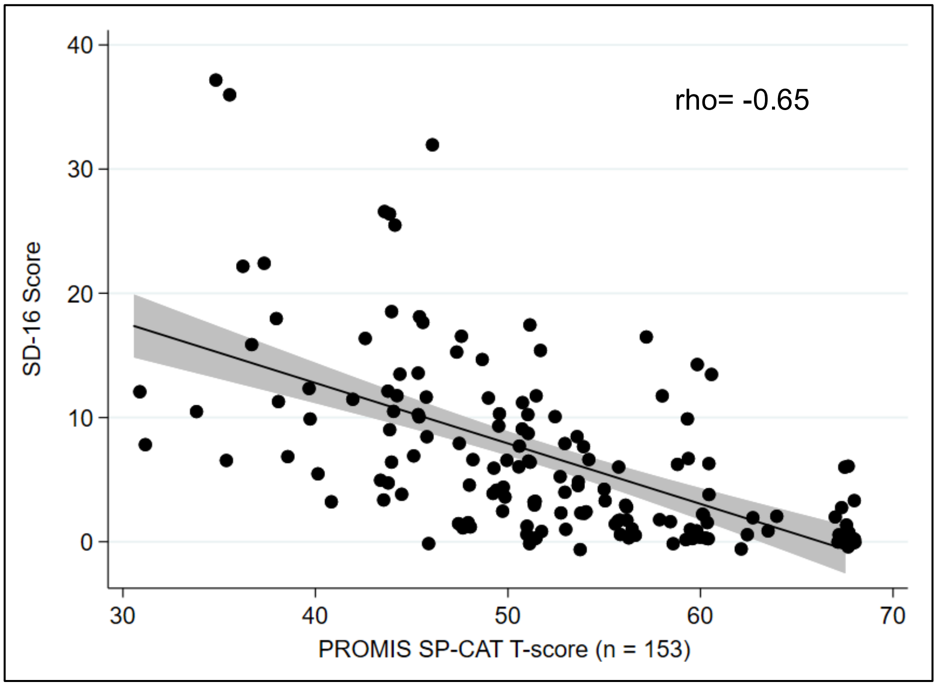

4.b

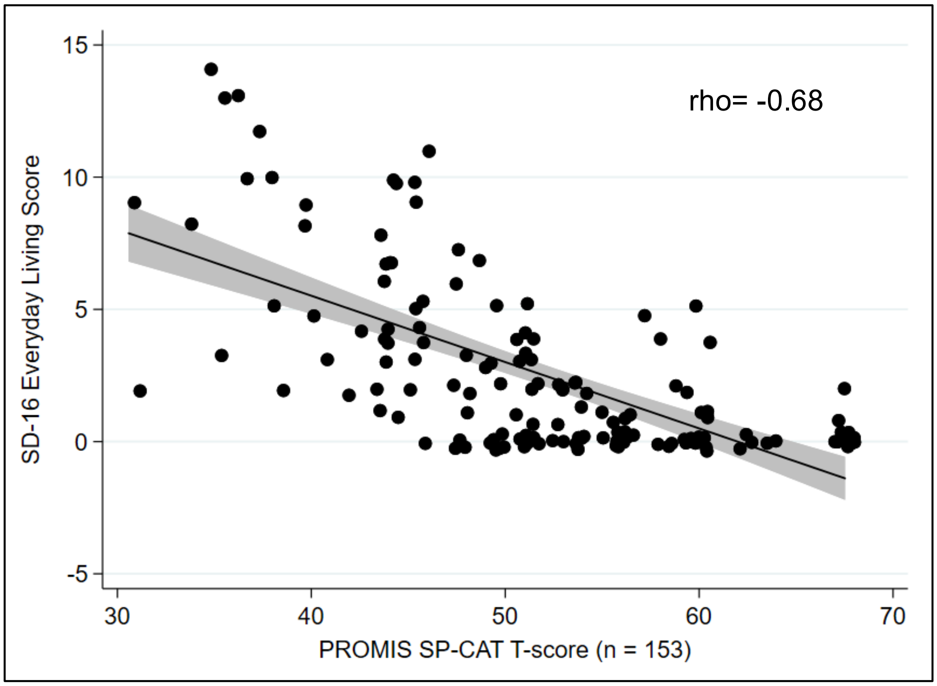

Footnote: Abbreviations: CAT, computer adaptive test; PROMIS, Patient-Reported Outcomes Measurement Information System; SP, social participation; SD-16, 16-item social distress.

Figure S5: Box plot between PROMIS-SP CAT T-scores and KDQOL-36 question 12* score for the whole sample.

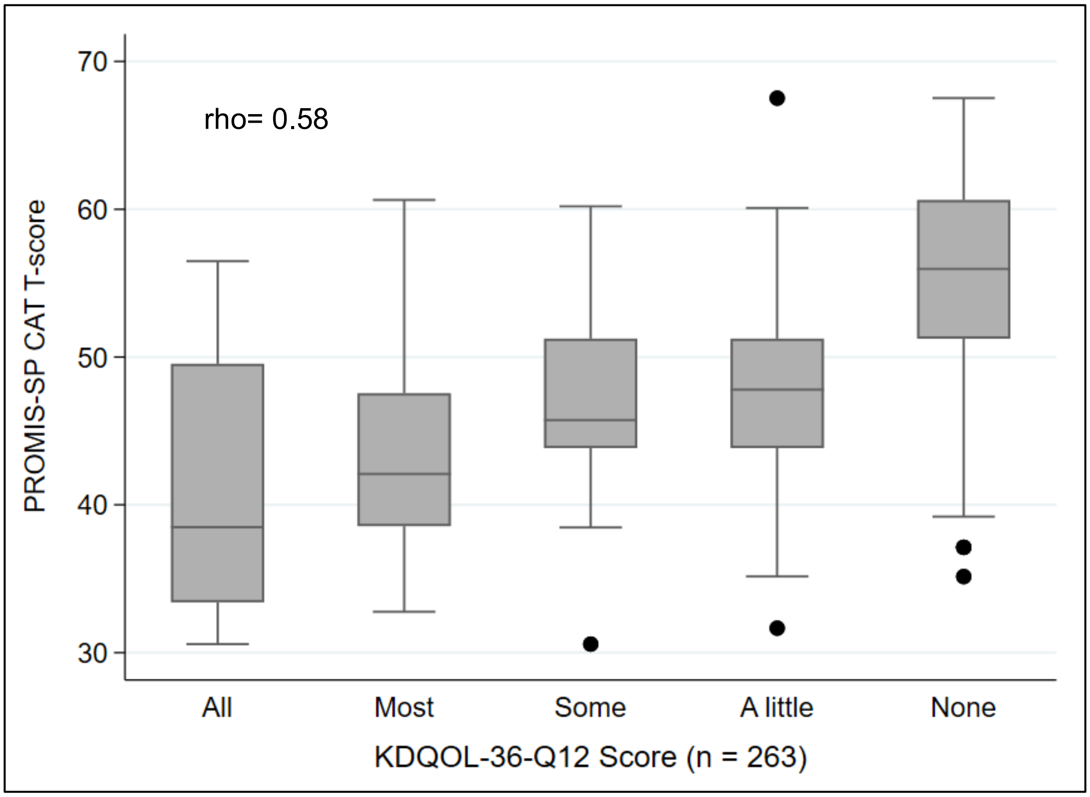

Footnote: The boxes show the interquartile range (IQR) around the median (horizontal line), while the whiskers extend to 1.5 times the IQR. Dots represent outliers beyond this range. Abbreviations: CAT, computer adaptive test; PROMIS, Patient-Reported Outcomes Measurement Information System; SP, social participation; KDQOL-36, Kidney Disease Quality of Life Instrument-36 Item Short Form; Q12, question 12.
*KDQOL-36 Question 12: "During the past 4 weeks, how much of the time has your physical health or emotional problems interfered with your social activities [like visiting with friends, relatives, etc.]?"

Table S1: PROMIS-SP CAT T-score cut-offs to identify kidney transplant recipients with no vs. significant social distress with SD-16 as a reference.

PROMIS SP-CAT T-score cut-offs	Sensitivity	Specificity	Youden's J index
40	24%	95%	0.20
42	29%	95%	0.24
44	47%	89%	0.36
46	62%	87%	0.49
47	62%	87%	0.49
48	67%	81%	0.47
**49**	**71%**	**80%**	**0.51**
**50**	**76%**	**74%**	**0.50**
51	87%	60%	0.47
52	87%	60%	0.47
53	88%	55%	0.44
54	89%	47%	0.36
55	89%	44%	0.33
56	89%	35%	0.24

Footnote: Abbreviations: CAT, computer adaptive test; PROMIS, Patient-Reported Outcomes Measurement Information System; SP, social participation; SD-16, 16-item social distress.

Table S2: Frequency of administration, response tendency, and mean t-score of respondents of last PROMIS-SP CAT items administered

PROMIS Social Participation item	Most common response (%)	Frequency of administration (n)	Mean T-score of respondents
I have trouble doing everything for my friends that I feel I should do.	Never (100%)	28	68
I have trouble doing all of the activities with friends that I feel I should do.	Never (100%)	2	64
I have trouble meeting the needs of my friends.	Never (91%)	2	62
I have to do my work for shorter periods of time than usual (include work at home).	Never (92%)	2	62
I have trouble participating in recreational activities with others.	Never (83%)	6	60
I have trouble doing all of the work that I feel I should do (include work at home).	Never (89%)	8	60
I have trouble doing everything for my friends that I want to do.	Never (79%)	5	59
I have trouble doing all the leisure activities with others that I want to do.	Never (91%)	10	57
I have to limit the things I do for fun with others.	Never (57%)	26	57
I have to limit my regular family activities.	Rarely (40%)	47	51
I have trouble doing all of the activities with friends that I want to do.	Never (51%)	17	50
I have trouble doing all of the family activities that I want to do.	Rarely (42%)	27	47
I have to limit my regular activities with friends.	Sometimes (41%)	71	46
I have trouble doing all of the work that is really important to me (include work at home).	Usually (50%)	11	38
I have trouble doing all of the family activities that I feel I should do.	Always (50%)	5	34
I have trouble doing all of the activities with friends that are really important to me.	Usually (100%)	1	32
I have trouble taking care of my regular personal responsibilities.	Sometimes (100%)	2	31

Table S3: Frequency of responses to last-items administered based on T-score range

		Last Item Administered				
**T-Score Range**		***I have to limit my regular family activities***	***I have trouble doing all of the family activities I want to do***	***I have to limit my regular activities with friends***	***I have trouble doing all of the activities with friends I want to do***	***I have to limit things I do for fun with others***
	30-39		Always = 3<br>**Usually = 6**<br>Sometimes = 1		Always = 3<br>**Usually = 5**	
	40-49	Always = 1<br>Usually = 1<br>**Sometimes = 17**<br>Rarely = 12	Usually = 2<br>**Sometimes = 10**<br>**Rarely = 10**	Usually = 6<br>**Sometimes = 15**<br>Rarely =6	Usually = 1<br>**Sometimes = 13**<br>Rarely = 3	Always = 1<br>Usually = 1<br>Sometimes = 2<br>**Rarely = 3**<br>Never = 2
	50-59		**Rarely = 7**<br>Never = 2	**Rarely = 8**<br>Never = 2	Sometimes = 3<br>Rarely = 4<br>**Never = 12**	Always = 1<br>Sometimes = 2<br>Rarely = 1<br>**Never = 12**
	≥60			Rarely = 1<br>**Never = 17**		Rarely = 1<br>**Never = 17**

Footnote: The table reports only the most representative and frequently used items, to allow for examination of differences in response patterns

STROBE Statement—Checklist of items that should be included in reports of ***cross-sectional studies***

	**Item No.**	**Recommendation**	**Pages**
**Title and abstract**			
	1	(*a*) Indicate the study's design with a commonly used term in the title or the abstract	1
		(*b*) Provide in the abstract an informative and balanced summary of what was done and what was found	2
**Introduction**			
Background/rationale	2	Explain the scientific background and rationale for the investigation being reported	4-5
Objectives	3	State specific objectives, including any prespecified hypotheses	5
**Methods**			
Study design	4	Present key elements of study design early in the paper	6-7
Setting	5	Describe the setting, locations, and relevant dates, including periods of recruitment, exposure, follow-up, and data collection	6-7
Participants	6	(*a*) Give the eligibility criteria, and the sources and methods of selection of participants	6-7
Variables	7	Clearly define all outcomes, exposures, predictors, potential confounders, and effect modifiers. Give diagnostic criteria, if applicable	6-11
Data sources/ measurement	8*	For each variable of interest, give sources of data and details of methods of assessment (measurement). Describe comparability of assessment methods if there is more than one group	6-11
Bias	9	Describe any efforts to address potential sources of bias	-
Study size	10	Explain how the study size was arrived at	-
Quantitative variables	11	Explain how quantitative variables were handled in the analyses. If applicable, describe which groupings were chosen and why	9-11
Statistical methods	12	(*a*) Describe all statistical methods, including those used to control for confounding	9-11
		(*b*) Describe any methods used to examine subgroups and interactions	9-11
		(*c*) Explain how missing data were addressed	9
		(*d*) If applicable, describe analytical methods taking account of sampling strategy	-
		(*e*) Describe any sensitivity analyses	-
**Results**			
Participants	13*	(a) Report numbers of individuals at each stage of study—eg numbers potentially eligible, examined for eligibility, confirmed eligible, included in the study, completing follow-up, and analysed	11
		(b) Give reasons for non-participation at each stage	11
		(c) Consider use of a flow diagram	*Supplemental Figure S1*
Descriptive data	14*	(a) Give characteristics of study participants (eg demographic, clinical, social) and information on exposures and potential confounders	11
		(b) Indicate number of participants with missing data for each	-

		variable of interest	
Outcome data	15*	Report numbers of outcome events or summary measures	-
Main results	16	(*a*) Give unadjusted estimates and, if applicable, confounder-adjusted estimates and their precision (eg, 95% confidence interval). Make clear which confounders were adjusted for and why they were included	11-13
		(*b*) Report category boundaries when continuous variables were categorized	10
		(*c*) If relevant, consider translating estimates of relative risk into absolute risk for a meaningful time period	n/a
Other analyses	17	Report other analyses done—eg analyses of subgroups and interactions, and sensitivity analyses	11-13
**Discussion**			
Key results	18	Summarise key results with reference to study objectives	13-14
Limitations	19	Discuss limitations of the study, taking into account sources of potential bias or imprecision. Discuss both direction and magnitude of any potential bias	17
Interpretation	20	Give a cautious overall interpretation of results considering objectives, limitations, multiplicity of analyses, results from similar studies, and other relevant evidence	14-17
Generalisability	21	Discuss the generalisability (external validity) of the study results	17
**Other information**			
Funding	22	Give the source of funding and the role of the funders for the present study and, if applicable, for the original study on which the present article is based	18

*Give information separately for exposed and unexposed groups.

Note: An Explanation and Elaboration article discusses each checklist item and gives methodological background and published examples of transparent reporting. The STROBE checklist is best used in conjunction with this article (freely available on the Web sites of PLoS Medicine at http://www.plosmedicine.org/, Annals of Internal Medicine at http://www.annals.org/, and Epidemiology at http://www.epidem.com/). Information on the STROBE Initiative is available at www.strobe-statement.org.
